# Different Retinoid Micellar Formulations on Wound Healing: Efficacy and Collagen Structure

**DOI:** 10.3390/pharmaceutics18060708

**Published:** 2026-06-09

**Authors:** David O. Oluwole, Robert Lees, Sneha Banerjee, Will Buchanan, Lian X. Liu

**Affiliations:** 1 School of Chemistry and Chemical Engineering, University of Surrey, Guildford GU2 7XH, UK; 2 Central Laser Facility, Science and Technology Facilities Council, Rutherford Appleton Laboratory, Harwell Science and Innovation Campus, Oxfordshire OX11 0QX, UK; 3 Phytoceutical Ltd., West Sussex GU29 9NQ, UK

**Keywords:** collagen, wound healing, 3D skin model, retinoids, retinol, retinaldehyde, retinoic acid, micelles

## Abstract

**Background**: The formation of wounds or scars often compromises skin anatomy and function, necessitating effective management to restore tissue integrity. Current interventions, including wound debridement, hyperbaric oxygen therapy, antibiotics, wound dressings, and surgical procedures, can be effective but are sometimes limited by high costs and the increasing prevalence of drug resistance. These challenges highlight the need for innovative, cost-effective, and therapeutic alternatives. **Method**. Our earlier studies assessed the wound closure of wounded human-equivalent epidermal full-thickness skin model (HEFT-SM) with a limited number of Phytoceutical^®^ retinol micellar formulations for a six-day treatment and found that the retinol micellar formulation accelerated wound closure significantly. In this work, three different types of Phytoceutical^®^ retinoid formulations, namely 0.3% retinol, 0.3% retinaldehyde, and 0.03% retinoic acid on the early-stage wound healing efficacy and its collagen structure were studied. Haematoxylin and eosin (H&E) staining analysis was used to assess the wound repair of the 3 mm punch wound after two days and the wound healing efficacy defined as the wound diameter contraction in percentage was assessed. The collagen matrix was examined through the use of Masson’s trichrome staining and confocal laser scanning microscopy (CLSM) for both qualitative and spatial assessment. **Results**. All formulations promoted wound contraction, with efficacy ranging from 15 ± 1% to 35 ± 2% after two days. The 0.3% retinol micelles showed the highest activity (35 ± 2%), followed by retinaldehyde (32 ± 3%) and retinoic acid (15 ± 1%). In addition, all treatments appeared to stimulate collagen architectural changes suggestive of remodelling activity. **Conclusions**. The enhanced wound healing observed may be attributed to increased cellular proliferation and migration within the wound microenvironment, supporting epidermal differentiation and tissue stratification. Furthermore, this work showed that combination of Masson’s trichrome staining and confocal laser scanning microscopy (CLSM) is a novel approach for qualitative and spatial assessment of collagen structure.

## 1. Introduction

Healthy normal wound repair involves coordinated processes including platelet activation (haemostasis), inflammation regulation, cellular proliferation, and extracellular wound matrix (ECM) generation and remodelling [1,2,3]. Collagen, constituting circa 25% of the protein content in mammals and the main protein product in fibroblast, plays a vital role in the ECM [3,4,5]. These functions include supporting molecular and cellular activities essential for keratinocytes and endothelial cells as well as fibroblasts migration at the wound site, improving native collagen synthesis for wound repair by restructuring tissue anatomy [3,4]. Connective tissues produce ECM including collagen I as part of the healthy wound healing process in response to compromised skin anatomy [3,4,5,6]. However, when ECM production becomes excessive, it can lead to tissue anatomical disorders such as keloids and hypertrophic scars [3,6,7,8]. Moreover, when wound healing is delayed, it can result in scar formation [3]. Collagen production during wound healing is crucial for maintaining tissue tensile strength and proper anatomical structure. Uncontrolled collagen synthesis or delays in wound repair can lead to various types of scars, including atrophic, keloid, or hypertrophic scars [3,6,7,8]. Specifically, collagen production in keloids and hypertrophic scars demonstrate an approximate twenty-fold and three-fold increase in collagen deposition, respectively, when compared to the typical physiological levels observed in healthy normal tissue [3,6,7,8]. Excessive scarring not only impairs physical function but also affects a patient’s psychological well-being [3]. In the developed world, circa 100 million patients develop scars from around 55 million elective operations and 25 million trauma-related procedures, with hypertrophic scarring incidence ranging from 40 to 70% after surgery and up to 91% following burn injuries, depending on wound depth [3,9,10].

Cofactors such as retinoids, ascorbic acid, and zinc have been shown to regulate collagen production, which is essential for ECM synthesis [3,11]. Prior studies have shown that retinoids can regulate collagen I and III expression resulting in elevated procollagen I and III levels promoting epidermal thickness and reduced facial wrinkles possibly due to well-modulated collagen [12,13]. In addition, previous research by our group demonstrated the wound closure efficacy of retinol-based micelle formulations against wounded human-equivalent epidermal full-thickness skin model (HEFT–SM) specimens after six days of treatment [14]. However, these studies did not address the activity of various retinoid micelle formulations on the HEFT-SM samples prior to the six-day treatment period. Moreover, some of the underlying mechanisms of the repair were not accounted for including wound ECM such as collagen generation. Sequel to this, our group was motivated to examine the wound contraction and healing of the wounded HEFT-SM samples following the two-day treatment period with three distinct Phytoceutical^®^ formulations, separately incorporating 0.3% retinol, 0.3% retinaldehyde, and 0.03% retinoic acid, and we elucidate the effect of the collagen in this context [14]. Furthermore, research into the efficacy of other retinoid-based micelle formulations including retinaldehyde and retinoic acid are rare. These retinoids have known roles in cell differentiation, proliferation, and collagen synthesis, but their specific impact on wound healing and tissue repair in comparison to retinol has not been fully elucidated [13,15,16,17].

Retinoids play a critical role in skin repair through their ability to modulate keratinocyte proliferation and migration, thereby enhancing epidermal turnover and stimulating collagen synthesis via retinoic acid receptor (RAR)-mediated pathways. Despite their widespread application in dermatology and cosmetic science, the role of different retinoid derivatives in wound healing remains complex, particularly when comparing precursor molecules such as retinol and retinaldehyde with the biologically active retinoic acid. Several studies have demonstrated that retinoic acid exhibits the highest intrinsic biological activity due to its direct interaction with nuclear receptors, whereas retinol and retinaldehyde require metabolic conversion to exert comparable effects. However, these studies largely evaluate retinoids in isolation and do not account for the influence of delivery systems on their biological performance. Recent advances have therefore focused on nanocarrier and micellar delivery systems to improve retinoid physicochemical properties, including solubility, stability, and skin penetration. For example, prior work using human epidermal full-thickness skin models (HEFT-SM) has shown that retinol-based micellar formulations significantly accelerate wound closure compared to untreated controls, underscoring the importance of formulation strategy in modulating therapeutic efficacy. In addition, comprehensive reviews of retinoid delivery systems highlight that encapsulation can enhance bioavailability while reducing irritation, a major limitation of conventional retinoid therapy. In contrast, most studies to date focus on single retinoid systems, and direct head-to-head comparisons of retinol, retinaldehyde, and retinoic acid within equivalent delivery platforms remain limited, representing a critical gap in the field [13,14,15,16,17]. While the retinoid active ingredients are the principal focus of this study, the contribution of the micellar carrier is of importance. Micelles are delivery systems capable of modifying retinoids’ physicochemical properties such as size, surface charge, critical micelle concentration, and interfacial dynamics. These properties can influence transdermal permeation and the local tissue microenvironment. Previous reports have demonstrated the importance of micellar systems in improving active ingredients’ physicochemical properties [14,17,18,19].

The techniques for measuring the properties of the recovered wound including the types and structure of the collagen produced are limited. A number of techniques including immunohistochemical assays via haematoxylin and eosin (H&E) staining have been employed to evaluate wound contraction. Conversely, immunohistochemical staining including van Gieson and Masson’s trichrome, alongside H&E staining, and microscopy (transmission electron microscopy and confocal laser scanning microscopy) have been leveraged to investigate collagen generation in both in vitro and ex vivo models. Numerous studies have shown that quantitative tissue assessment techniques, particularly advanced imaging modalities such as confocal laser scanning microscopy, could provide vital insights into tissue repair mechanisms during the wound healing process [20,21]. Moreover, biochemical quantification methods such as hydroxyproline and RT-qPCR could be applied in assessing bulk quantification of the collagen; however, our current study focuses on determining the spatial and structural collagen organisation using Masson’s trichrome staining and CLSM.

Herein, we investigated three distinct Phytoceutical^®^ formulations, containing 0.3% retinol, 0.3% retinaldehyde, and 0.03% retinoic acid, for their impact on wound healing and collagen production, a critical factor in epidermal differentiation and tissue stratification. Our investigation employs HEFT-SM specimens, treated for two days (48 h), with subsequent analysis conducted using H&E staining. The qualitative elucidation of the collagen morphology of the (un)treated HEFT-SM specimens was determined via Masson’s trichrome staining and confocal laser scanning microscopy. The HEFT skin model served as a suitable in vitro model due to its capacity to mimic human skin physiology and wound healing processes. This model has proven to be a relevant tool for evaluating the efficacy of new materials designed for wound repair [22]. In addition, we report for the first time the dual deployment of Masson’s trichrome staining for structural collagen assessment alongside CLSM-based Fast Green (FG) fluorescence labelling for spatially resolved, three-dimensional collagen mapping.

Furthermore, the concentrations of retinol and retinaldehyde utilised were tenfold greater than that of retinoic acid, owing to retinoic acid’s twenty-fold higher potency compared to retinol and retinaldehyde [17]. The selected doses (0.3% retinol, 0.3% retinaldehyde, 0.03% retinoic acid) were based on approximate potency equivalence. However, biological equivalence is not absolute due to differences in metabolism, receptor activation, and bioavailability.

## 2. Materials and Methods

Benzyl alcohol, benzyl benzoate, absolute ethanol (99.8%), methanol (HPLC grade), Fast Green FCF, and ethyl cinnamate were obtained from Sigma Aldrich (St. Louis, MO, USA) without further purification. Cultures of HEFT-SM (EpiDermFT-400), serum-free maintenance media, assay media, phosphate-buffered saline solution (PBS), and 6-well tissue culture plates were procured from MatTek^®^, Mlynske Nivy, Slovak Republic. Fisherbrand™ (Loughborough, UK) Multi-Platform Shaker was used during tissue optical clearing. Leica SP8 microscope (Milton Keynes, UK) was used to elucidate collagen fibril orientation and architecture of the human-equivalent skin samples. The retinoid-based micellar formulations (RMF) were made following the Phytoceutical Ltd. IP protocol (Br. Pat. GB2550346 and Eur. Pat. App. 17724421.7). Detail protocol for the micellar formulation preparation has been provided in the Supplementary Materials Section.

### 2.1. Treatments

Treated and untreated EFT tissue samples were separated randomly into three experimental groups: punch-wounded, UV-wounded and unwounded HEFT-SM tissues (control; no formulation was administered except for PBS to keep the tissue apical surface moist). Intact HEFT-SM specimens’ irradiation was conducted at 366 nm for 25 min (fluence: ~3.4 J/cm^2^). The treated HEFT-SM tissues were exposed to 25 µL of a single formulation on the apical surface to ensure uniform surface coverage of the tissue model. The formulations being tested were 0.3% retinol micelles (F1), 0.3% retinaldehyde micelles (F2), and 0.03% retinoic acid micelles (F3). The treated and untreated HEFT-SM cultures in 6-well tissue culture plates were maintained and assayed in a humidified incubator (95% relative humidity) at ~37 °C with ~5% CO_2_ (Thermo Fisher^®^, Waltham, MA, USA). The HEFT-SM consists of the epidermal layer containing stratum corneum and keratinocytes, and dermis composed of fibroblasts alongside deposited collagen matrix. The specimens were maintained in maintenance or assay media provided by the tissues manufacturer with replacement of the basal compartment media at predetermined time intervals. After the treatment period, the tissues were carefully removed from the membrane inserts with tissues stored in a 4% paraformaldehyde (PFA) methanolic solution for histological and confocal microscopy analysis.

### 2.2. Wound-Contraction Measurement

**Figure 1:**
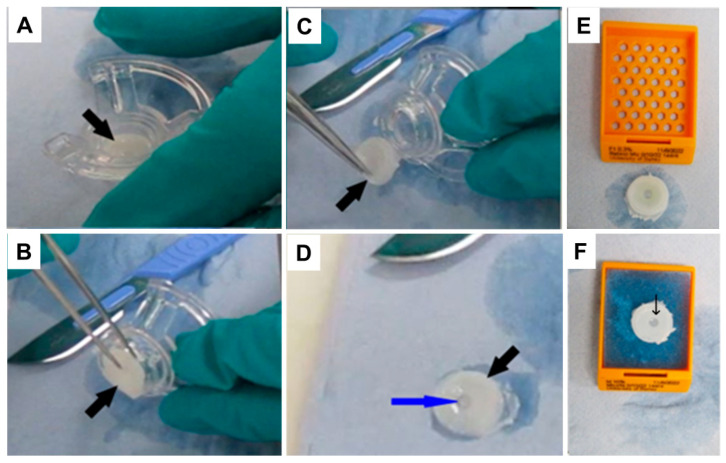
Representative macrographs demonstrating the removal (**A**–**D**) of the HEFT-SM specimens from the insert wells following treatment. (**D**–**F**) exhibit the morphology of the HEFT-SM tissues. The black arrow depicts the HEFT-SM specimens; the blue arrow indicates the punch wound.

Figure 1A–D show the representative macrographs depicting the removal of the HEFT-SM specimens from the insert wells following treatment.

Haematoxylin and eosin (H&E)-staining analysis was used to assess the wound repair of the 3 mm punch wound. The treated and untreated wounded HEFT-SM were stored in 4% PFA and followed by gross evaluation of the tissues texture as shown in Figure 1A–F. Thereafter, they were routinely processed, embedded in paraffin-wax, and blocks were made by surface flattening wounded HEFT-SM specimens to obtain homogeneous histological sections. Additionally, 5 μm thick sections were created and stained with H&E. The sectioned samples were scanned on a calibrated NanoZoomer Digital slide scanner (Hamamatsu^®^, Hertfordshire, UK). Digital measurements of the diameters of the wounded HEFT-SM specimens were conducted horizontally [14].

### 2.3. Masson Trichrome Staining

For the collagen detection using histological assay [23], Masson’s trichrome staining (using a ready-to-use kit) was employed to qualitatively assess collagen production in response to the retinoid micellar formulations applied to the HEFT-SM specimens, following established protocols with minor modifications. The 4% PFA fixed HEFT-SM samples were deparaffinised, hydrated and sectioned. Thereafter, they were immersed in Bouin’s solution for 15 min to re-fix the HEFT-SM specimens. After fixation, the samples were treated with Weigert’s hematoxylin for 5 min, followed by Biebrich scarlet acid fuchsin staining for another 5 min. The tissues were subsequently differentiated by immersion in a phosphotungstic–phosphomolybdic acid solution for 5 min to enhance collagen staining. Finally, the differentiated HEFT-SM samples were stained with aniline blue solution to specifically stain the collagen fibre blue, enabling clear visualisation and evaluation of the collagen fibril orientation and arrangement.

### 2.4. Clearing and Staining

The (un)treated HEFT-SM tissues were cleared, stained and imaged by confocal laser scanning microscopy using adapted protocols previously described by Abadie et al. and Milinkovitch et al. [20,21]. The (un)treated HEFT-SM samples were washed with PBS for 1 h to remove any residual formulation, followed by overnight immersion in 4% paraformaldehyde (PFA) in PBS at room temperature with agitation on a shaker at 120 rpm. Afterwards, the samples were washed with PBS (3 times, 1 h each) at ambient temperature with agitation on a shaker at 120 rpm. The tissues were then immersed in methanol-in-PBS solutions of increasing concentrations (30%, 50%, 70%, and 100% methanol; 1 h each) with agitation on a shaker at 120 rpm. Finally, the tissues were immersed in 100% methanol overnight.

The fully dehydrated tissues were immersed in 2 μg/mL of Fast Green FCF (FG; Thermo Scientific) in methanol for 5 days and maintained on a shaker.

The FG-stained tissues were incubated in one part benzyl alcohol and two parts benzyl benzoate (BABB) solution and maintained at ambient temperature for 2 days on a shaker. The cleared FG-stained tissues were transferred into ethyl cinnamate and imaged using confocal microscopy.

### 2.5. Confocal Laser Scanning Microscopy

Volume images of cleared tissue samples were acquired using a Leica SP8 confocal laser scanning microscope equipped with a white light laser (NKT SuperK Extreme). The sample was placed in a commercially available Leica sample mounting frame (Leica, 158007063, Milton Keynes, UK) with #1.5 cover glass cemented in place with silicone sealant (Wacker E43, Milton Keynes, UK). The mounting frame was filled with Ethyl cinnamate so that the sample was covered but not floating or moving. A 25x/0.95 NA objective was used for excitation and detection, with a final lateral pixel dimension of 0.3 micrometres.

The surface of the sample was acquired using mosaic tiling and stitched together afterwards in the Leica LAS X software. The entire depth of the sample was acquired by creating stacks of images 20 um apart. Wavelengths used for imaging were 488 nm (autofluorescence) and 627 nm (Fast Green FCF). Filters were placed before the camera to capture each wavelength—a bandpass at 500–605 for 488 nm and 635–760 nm for 627 nm illumination.

All samples were imaged using identical detector settings throughout the study, and detector gain was maintained constant across all experimental groups during image acquisition to ensure consistency in fluorescence intensity measurements. Furthermore, excitation adjustments applied during z-stack acquisition to compensate for signal attenuation caused by tissue scattering at increasing imaging depths were performed systematically and identically across all samples. No photobleaching correction was applied during image analysis because fluorescence quantification was derived from single-frame acquisitions at each imaging plane rather than from time-lapse imaging experiments. Under these acquisition conditions, photobleaching effects are expected to be negligible and were minimised through standardised imaging procedures applied uniformly across all samples.

### 2.6. Image Analysis

Fiji ImageJ was used to visualise the volumetric data acquired from the Leica SP8 confocal microscope. To enhance image clarity and highlight collagen fibril orientation and structural architecture, the enhance local contrast (CLAHE) tool was applied. This method improved the local contrast in the images by using specific settings including block size (50), bins (256), and max slope (2.5). These parameters were selected based on literature to enhance image quality, resulting in better image visualisation of HEFT-SM tissue structures with the identification of important features including collagen fibril orientation and organisation [24].

### 2.7. Statistical Analysis

All experiments were conducted using independent replicates (n = 3), with each condition tested in triplicate to ensure reproducibility. Data are presented as mean ± standard error of the mean (SEM). Statistical comparisons between multiple groups were conducted using one-way analysis of variance (ANOVA) to evaluate overall differences across treatments. Where significant effects were identified, appropriate post hoc multiple comparison tests were applied to determine pairwise differences between groups. A two-tailed *p*-value of less than 0.05 (*p* < 0.05) was considered statistically significant.

## 3. Results

### 3.1. Wound Repair Investigated by H&E Staining

Figure 1D–F depict the representative macrographs showing the morphology of the HEFT-SM specimens.

The macroscopic analysis of the HEFT–SM samples exhibited mild variation including the central to para-centre punched wound per manufacturer as shown in Figure 1D–F.

**Figure 2:**
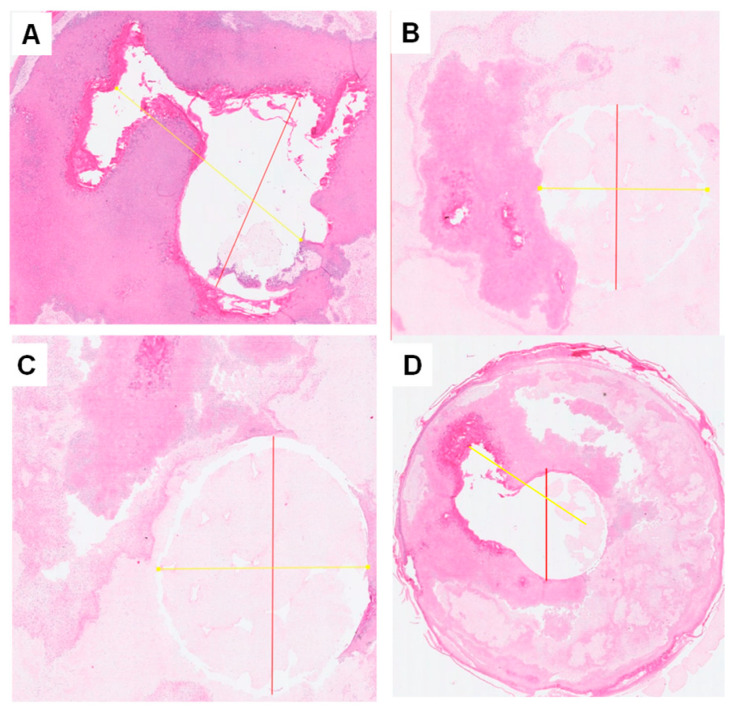
Representative photomicrographs of punch-wounded HEFT-SM specimens’ wound closure after treatment with retinoid-based micellar formulations (RMF) compared to the control measured using H&E staining after the two-day treatment period. (**A**). Control; (**B**). 0.3% retinol micelles (F1); (**C**). 0.3% retinaldehyde micelles (F2); (**D**). 0.03% retinoic acid (F3).

Figure 2A–D show the representative photomicrographs of RMF-treated wounded HEFT-SM specimens’ wound closure, with analysis conducted via H&E staining following the 48 h treatment period.

The photomicrograph analysis of the RMF-treated wounded HEFT-SM specimens showed improved wound closure compared to the control groups (placebo). This is demonstrated through the reduction in the wound diameter due to rapid wound closure driven by the formulations indicating wound healing potential of the RMF as shown in Figure 2.

Quantification of the H&E-stained photomicrographs analysis of the punched wound was conducted, and the data are presented as the wound diameter reduction, expressed as follows [14].

$$\%\;WC=\frac{Initial\;wound\;diameter-WD\;after\;2\;days}{Initial\;wound\;diameter}\times 100$$

**Figure 3:**
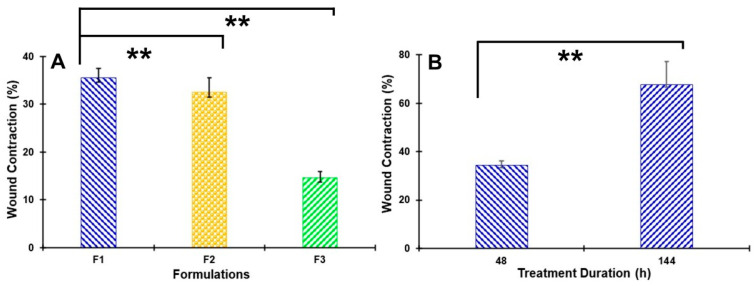
Wound closure of full-thickness human ex vivo skin (HEFT-SM) following treatment with retinoid-based micellar formulations, quantified from H&E-stained sections. (**A**) Percentage wound closure after 48 h of treatment with 0.3% retinol micelles (F1), 0.3% retinaldehyde micelles (F2), and 0.03% retinoic acid micelles (F3), compared to untreated control. (**B**) Percentage wound closure for F1-treated specimens across two treatment durations—48 h (present study) and 6 days ([14])—to illustrate temporal progression of retinol-mediated wound closure. ** *p* < 0.05. Data are expressed as percentage wound closure relative to the 0 h baseline and presented as mean ± SEM (n = 3).

Here, % WC = wound diameter contraction measured in percentage and WD = wound diameter of the treated HEFT-SM with either PBS or different formulations. The initial wound diameter is 3 mm (the original inner wound diameter of the HEFT-SM from the supplier). The percentage of wound contraction is shown in Figure 3.

Figure 3 shows the quantification of wounded HEFT-SM specimens’ wound closure following treatment with retinoid-based micellar formulations, with analysis conducted using H&E staining.

### 3.2. Collagen Assay by Masson’s Trichrome Staining (MTS) and Confocal Laser Scanning Microscopy (CLSM)

The HEFT-SM dermis layer is primarily reminiscent of papillary and reticular dermis, which is characterised by a loose meshwork of thin fibromyxoid stroma with pre-deposited collagen consisting of a delicate branching network of fine elastic fibres [25,26]. MTS and FG-labelled CLSM technique data were interpreted based on changes in collagen fibre morphology including arrangement and orientation rather than absolute collagen presence and content.

#### 3.2.1. Collagen Assay by Masson’s Trichrome Staining

**Figure 4:**
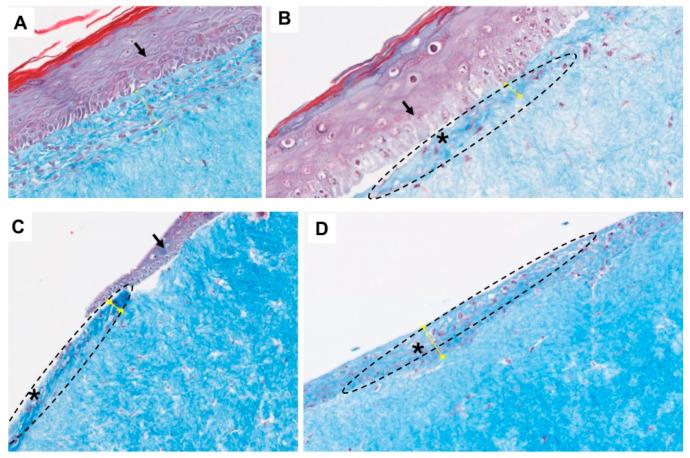
Representative MTS photomicrographs of wounded HEFT-SM specimens. (**A**). Control, (**B**). specimens treated with 0.3% retinol micelles, (**C**). specimens treated with 0.3% retinaldehyde micelles, and (**D**). specimens treated with 0.03% retinoic acid micelles. Asterisks highlight possible collagen architectural changes. Specifically, this pattern was more pronounced at the connective tissues of the RMF-treated specimens; see Figure 4B–D (dotted circle).

Figure 4 demonstrates the representative Masson’s trichrome staining (MTS) photomicrographs of wounded HEFT-SM specimens.

Masson’s trichrome staining was used to qualitatively assess the collagen deposition and organisation in the specimens. While quantitative photomicrograph analysis can provide data on collagen abundance, MTS qualitative technique was used to gain insight into the spatial distribution of the collagen fibres before and after treatment.

#### 3.2.2. Collagen Determination by Confocal Laser Scanning Microscopy (CLSM)

To visualise the effects of formulations on collagen production, Fast Green (FG) dye which binds to collagen-rich regions was applied to HEFT-SM specimens, and imaging was conducted using confocal laser scanning microscopy (CLSM). To improve light penetration and enable deeper tissue scanning, optical tissue clearing was incorporated into the preparation workflow [20,21]. Second harmonic generation (SHG) microscopy has previously served as a primary tool for elucidating the structural architecture and orientation of collagen fibrils. As a label-free, non-linear optical technique, SHG exploits the non-centrosymmetric molecular arrangement of fibrillar collagens to generate a coherent optical signal without exogenous dyes. Because the SHG signal arises exclusively from highly ordered, non-centrosymmetric structures, it is inherently selective for fibrillar collagen, providing quantitative information on fibre organisation, density, and directionality with high spatial resolution. Notably, SHG does not require optical tissue clearing. However, SHG has two principal limitations. First, refractive index mismatches within tissue restrict penetration depth, constraining the range of light scanning. Second, while SHG is selective for fibrillar collagen architecture, it does not distinguish between collagen subtypes or detect non-fibrillar collagen pools, limiting the breadth of collagen-related information it can provide. FG-labelled CLSM, combined with optical tissue clearing, addresses the penetration depth limitation and enables imaging of heterogeneous samples at greater tissue depths [20,21]. Nevertheless, an important interpretive caveat applies: Fast Green FCF is a general protein stain whose electrostatic binding under acidic conditions is not restricted to collagen but extends to a broad range of tissue proteins, including cytoplasmic and structural proteins present in both the extracellular matrix and cellular compartments. Fluorescence readouts obtained with FG-labelled CLSM may therefore overestimate collagen content where non-collagenous proteins co-localise within the imaged region. This risk is particularly relevant in wounded or treated specimens where elevated cellular density, inflammatory infiltrate, or active matrix remodelling may substantially alter the local protein composition. While SHG offers label-free specificity for fibrillar collagen architecture, FG-labelled CLSM provides greater penetration depth and sensitivity in heterogeneous tissues, albeit with reduced biochemical specificity [27,28,29,30].

Initially, collagen fibrils deposited in wounds often exhibit a disorganised arrangement, leading to poorly defined structures and textures. However, as wound repair advances, a well-defined orientation becomes evident [3].

**Figure 5:**
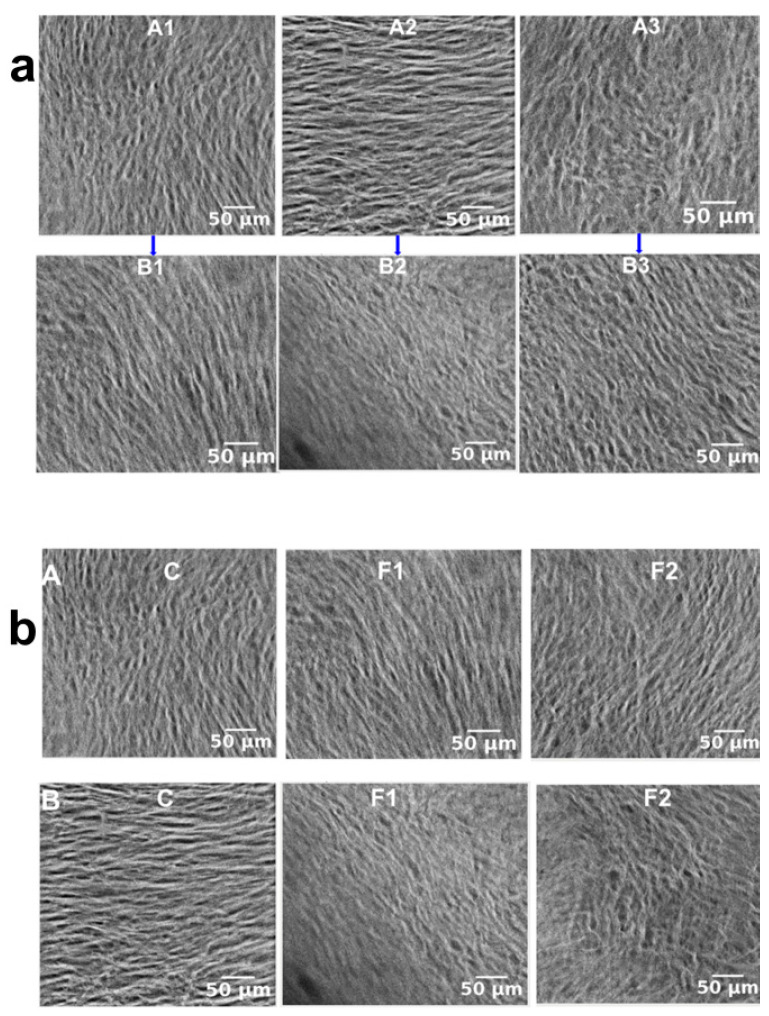
FG-labelled confocal laser scanning microscopy (CLSM) of collagen fibrils in the dermis of HEFT-SM specimens after 48 h of treatment. (**a**) Representative FG-labelled CLSM photomicrographs showing dermal collagen fibril architecture in untreated (control) and retinol micelle formulation (RMF)-treated HEFT-SM specimens across three different conditions: (1) unwounded skin, (2) punch-wounded skin, and (3) UV-irradiated skin. Panel A: untreated control; Panel B: RMF-treated. Scale bar: 50 µm. (**b**) Representative FG-labelled CLSM photomicrographs showing dermal collagen fibril architecture in unwounded and punch-wounded HEFT-SM specimens treated with retinoid micelle formulations. Panel C: untreated control; Panel F1: retinol micelle-treated; Panel F2: retinaldehyde micelle-treated. Scale bar: 50 µm.

Figure 5 illustrates the representative FG-labelled CLSM photomicrographs of collagen fibrils imaged at the dermis of the untreated and RMF-treated HEFT-SM specimens after the two-day treatment period.

The collagen fibrils’ orientation and structural architecture in both treated and untreated HEFT-SM specimens were investigated using FG-labelled confocal laser scanning microscopy imaging as shown in Figure 5.

**Figure 6:**
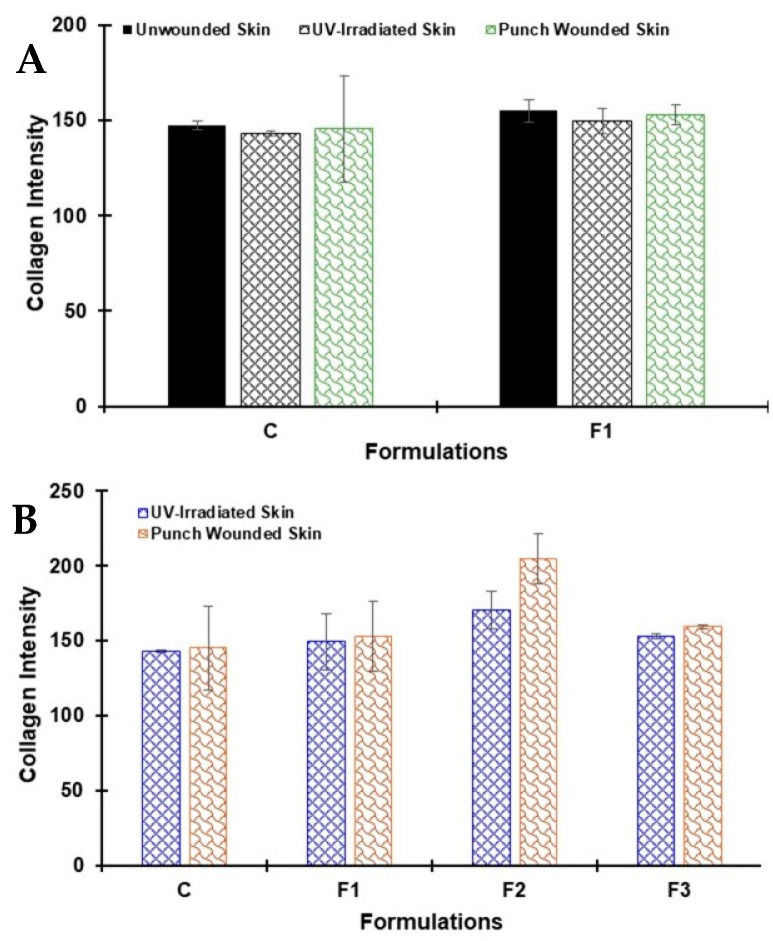
Quantitative analysis of collagen-associated fluorescence intensity in untreated (control) and retinoid micelle formulation (RMF)-treated HEFT-SM specimens. (**A**) Collagen fluorescence intensity in unwounded, punch-wounded, and UV-irradiated tissues comparing control and F1-treated (0.3% retinol micelles) groups. (**B**) Comparative collagen fluorescence intensity in punch-wounded and UV-irradiated tissues across control and all RMF-treated groups: F1 (0.3% retinol micelles), F2 (0.3% retinaldehyde micelles), and F3 (0.03% retinoic acid micelles).

Figure 6 depicts the chart showing the collagen fluorescence intensity of the untreated and treated HEFT-SM tissues.

The control HEFT-SM specimens without the retinoid-based micellar formulations were either wounded, UV-light irradiated (UV-wounded), or left intact (unwounded). These samples exhibited fluorescence typical of collagen. The fluorescence intensity in the control group was lower compared to tissues treated with the formulations (Figure 5a).

## 4. Discussion

Following two days of treatment, re-epithelialisation with a differentiated epidermal layer was observed in the RMF-treated HEFT-SM specimens compared to the control group, thereby accelerating wound closure as depicted in Figure 2A–D. This expedited wound closure may be attributed to retinoids (formulation) stimulating the wound microenvironment, leading to cellular proliferation and fibroblast migration at the wound site, which are crucial initial steps in wound repair. Moreover, the formulations could have promoted the extracellular matrix activation, keratinocyte recruitment, and fibroblast proliferation and migration activity at the wound site. Furthermore, all the treated HEFT-SM specimens demonstrated narrower wound diameters and significant wound contractions (the shortest distance across the wound site) within 48 h, in contrast to the control group (placebo). The observed diameters ranged from <1 to 35 ± 2%. The control group exhibited <1% wound contraction (WC), indicating no significant natural wound healing activity after 48 h treatment period. In contrast, formulation F1, containing 0.3% retinol micelles, showed a 35 ± 2% reduction. This was followed by F2 (retinaldehyde micelles) and F3 (retinoic acid micelles) with reductions of 32 ± 3% and 15 ± 1%, respectively. Among all the tested formulations, F1 demonstrated the highest WC at 35 ± 2%, while the lowest WC was observed with F3 at 15 ± 1%. This difference may be attributable to the applied low dose of the latter. The enhanced WC observed with RMF could be attributed to the cellular proliferation and migration, as well as epidermal differentiation and tissue stratification, driven by these formulations. Furthermore, the likely collagen architectural changes originating from the dermis may have contributed to the accelerated wound closure; see Figure 4 [4,31,32,33,34,35]. These results align with prior research conducted by our team [14] and other investigators, which has consistently demonstrated the effectiveness of bioactive formulations in promoting keratinocyte recruitment and migration, as well as fibroblast proliferation and migration. This process, initiated by collagen, leads to re-epithelialisation within the wound microenvironment, thereby accelerating wound closure [12,32,33]. The near-zero contraction in untreated controls at 48 h is consistent with our prior research where only 8% wound contraction was observed following a 6-day treatment [14]. Ex vivo skin models lack the fibroblast-populated contractile stroma and cytokine-driven cellular recruitment that underpin early wound contraction in vivo; passive contraction at 48 h in the absence of exogenous stimulation is therefore not unexpected and is consistent with reported behaviour in comparable models in the literature [36,37].

The MTS of the RMF-treated HEFT–SM specimens exhibited consistent improvement in the dermal collagen staining with enhanced tissue architecture in all the treated specimens compared to control group (placebo). The wounded HEFT–SM specimens were stained with MTS, with the photomicrographs displaying the epidermis in purple colouration, and dermis collagen in blue to light blue colouration. In addition, the MTS photomicrographs demonstrated improved collagen expression in the RMF-treated HEFT–SM specimens suggesting that RMF elicited ECM production and stratification (maturation) through the appearance of collagen during wound closure (Figure 4). Moreso, the emergence of MTS patterns with finer, less densely packed collagen fibres in the treated HEFT-SM specimens consistent with collagen architectural changes during wound repair was observed. The appearance and distribution of these collagen architectural changes in the treated HEFT-SM specimens suggest active collagen generation which is a crucial factor in wound healing and tissue remodelling; see Figure 4 [35]. The untreated wounded HEFT-SM specimens (control) exhibit sparsely organised dermal collagen with a homogeneous blue colouration (Figure 4A). In contrast, the treated HEFT-SM specimens demonstrate a well-organised collagen fibre distribution, characterised by heterogeneous blue-staining intensity at the periphery of the wound site (connective tissues); see Figure 4B–D. Collagen fibres are stained blue, and the circled area suggests the presence of collagen architectural changes suggestive of remodelling activity. Arrows denote the epidermis, and light blue indicates dermal collagen. Asterisks highlight possible collagen architectural changes; see Figure 4. Specifically, this pattern was more pronounced at the connective tissues of the RMF-treated specimens; see Figure 4B–D (dotted circle). The improved collagen deposition and appearance of possible collagen architectural changes was supported by enhanced fibre organisation, possibly indicating regenerative remodelling [3,5,32,33,34,35]. Moreover, retinoids have been reported to prevent collagen degradation with collagen fostering the migration of keratinocyte, a cell vital for re-epithelisation in the wound repair process [3,12,13,14,15,16].

For the FG-labelled CLSM collagen determination, two distinct wound conditions were employed to capture a broader spectrum of clinically relevant tissue responses: UV-irradiated skin and punch-wounded skin. UV irradiation of the ex vivo skin model was applied to induce a photodamage response. This included the generation of reactive oxygen species (ROS) and oxidative stress, leading to progressive collagen degradation and impairment of keratinocyte proliferation and differentiation. Collectively, these changes are characteristic of photoaged or chronically compromised tissue, making this model particularly relevant for simulating the wound microenvironment encountered in elderly or sun-damaged skin, where healing capacity is often diminished. In contrast, the punch-wound model was employed to simulate an acute, mechanically defined wound, characterised by a discrete zone of full-thickness tissue disruption. This model is widely used in wound healing research for its reproducibility and its capacity to elicit well-characterised reparative responses, including inflammatory cell infiltration, granulation tissue formation, and collagen remodelling at the wound margin [38,39].

In the FG-labelled CLSM photomicrographs, the untreated HEFT-SM specimens exhibited distinct patterns in collagen fibril orientation and density. In the unwounded (control) skin, collagen fibrils were vertically aligned and exhibited a less dense arrangement, reflecting the typical structure of healthy dermis (Figure 5a). In contrast, wounded HEFT-SM tissues displayed notable alterations in collagen fibril organisation. The punch-wounded samples exhibited a horizontal orientation with densely arranged collagen fibrils, consistent with early wound healing stages where fibroblasts actively migrate to the wound site and deposit a new extracellular matrix. Meanwhile, UV-wounded samples demonstrated highly cross-linked collagen fibrils, suggesting a disorganised repair response during the initial phases of healing (Figure 5a).

The collagen architecture in the treated HEFT-SM specimens presented a markedly different profile. In the unwounded tissues treated with retinol micelles, the collagen maintained a vertical orientation like the control group, though the fibrils appeared slightly denser, possibly indicating an effect of the treatment (Figure 5b).

In the retinol micelle-treated punch-wounded tissues, a vertical fibril orientation was observed alongside a less dense arrangement compared to the untreated punch wounds, suggesting that the treatment may modulate collagen deposition to promote a more organised repair. In addition, retinol micelle-treated UV-wounded samples revealed cross-linked collagen fibrils with a para-vertical orientation that were better organised than those in the untreated UV wounds. A similar pattern emerged with retinaldehyde micelles treatment, reinforcing the idea that both retinol and retinaldehyde can enhance collagen organisation during wound healing (Figure 5b). Comparing the treated and untreated EFT skin models, the unwounded tissues exhibited similar collagen fibril orientations and structural architectures, indicating that the treatments did not disrupt the baseline dermal structure (Figure 5a,b). However, in wounded tissues, treated samples revealed a more organised collagen fibril arrangement than their untreated counterparts. The presence of highly dense or excessively cross-linked fibrils in some samples may indicate dysregulated fibroblast migration or altered enzymatic activity responsible for collagen organisation, which could potentially lead to fibrosis or scar formation if not properly controlled [3]. Moreover, the variations in collagen orientation and arrangement between unwounded and wounded human skin equivalents may be attributed to the dynamic biochemical environment present during tissue repair [3]. The collagen fluorescence intensities in unwounded, UV-irradiated, and punch-wounded tissues were observed to increase in the tissues that were treated with the retinoid micellar formulations compared to the untreated control groups (using F1 as an example; Figure 6A). Moreover, comparative analysis of the untreated and RMF-treated UV-irradiated and punch-wounded specimens revealed that F1–F3 demonstrated higher fluorescence intensities when compared to the control, which suggests their potential in stimulating collagen production. Particularly, F2, which incorporated both retinaldehyde and TPGS (a vitamin E derivative), displayed the highest fluorescence intensity. This indicates that F2 may possess a more potent ability to stimulate collagen synthesis than the other formulations, as shown in Figure 5. This observed pattern could be attributed to retinaldehyde’s role as a metabolic precursor to retinoic acid in human keratinocytes, potentially leading to an accelerated stimulation of collagen compared to retinol [17]. Consequently, the fluorescence intensities of the treated samples were not very high, and this is consistent with the literature which suggests that net collagen production increase could take up to 4–5 weeks after wounding [3]. Retinoids are well-documented with known irritation and sensitisation risks, specifically retinoic acid, and the micellar delivery strategy employed here has direct relevance to mitigating such effects through controlled release and reduced free retinoid availability at the tissue surface due to the role of micellar encapsulation in modulating skin-penetration kinetics [13,15,16,17,18,19].

## 5. Conclusions

At applied doses of 0.3% retinol, 0.3% retinaldehyde, and 0.03% retinoic acid, all micellar retinoid formulations demonstrated rapid wound repair compared to the untreated controls. Moreover, retinol and retinaldehyde demonstrated strong efficacy and potential lower irritation compared to retinoic acid with known potent biological activity but comparatively lower performance at applied doses and likely higher irritation risk.

H&E staining confirmed accelerated re-epithelialisation, improved epidermal differentiation, and enhanced tissue stratification in treated HEFT-SM specimens. Masson’s trichrome staining and confocal laser scanning microscopy (CLSM) revealed preservation of collagen integrity with appearance of likely reorganised collagen deposition with well-organised fibrillar architecture, consistent with structured extracellular matrix remodelling. Quantitative and spatial analyses further confirmed elevated collagen production across all treatment groups at the tested concentrations. The application of FG-labelled CLSM to assess the collagen morphology of (un)treated HEFT-SM marks a novel use of this technique in the evaluation of collagen within organotypic tissues like HEFT-SM.

These findings underscore the therapeutic promise of TPGS-based micellar retinoid formulations for accelerating wound closure and enhancing collagen generation and organisation during wound repair. While the study demonstrates potential in preclinical applications, the sample size was limited, and the HEFT skin model, though physiologically relevant, does not fully replicate the complexities of in vivo wound healing, particularly systemic immune responses. Although relative equivalent applied doses (0.3% retinol, 0.3% retinaldehyde, and 0.03% retinoic acid) were chosen to reflect potency, receptor-level activation and intracellular conversion efficiency were not directly quantified. Furthermore, collagen assessment primarily utilised histochemical and imaging analyses; complementary biochemical quantification such as gene expression profiling would further bolster mechanistic interpretation. Additionally, irritation potential and inflammatory biomarkers were not thoroughly characterised, which is especially important when comparing retinoid derivatives. In sum, future in vivo validation and expanded mechanistic studies are necessary to confirm translational applicability.

## Data Availability

The original contributions presented in this study are included in the article and Supplementary Material. Further inquiries can be directed to the corresponding author.

## Supplementary Materials

The following supporting information can be downloaded at https://www.mdpi.com/article/10.3390/pharmaceutics18060708/s1.

## Abbreviations

The following abbreviations are used in this manuscript:

RMF	Retinoid-based micellar formulation
HEFT-SM	Human-equivalent epidermal full-thickness skin model
MTS	Masson trichrome staining
CLSM	Confocal laser scanning microscopy
TPGS	D-α-Tocopherol polyethylene glycol succinate
H&E	Haematoxylin and eosin staining
PBS	Phosphate-buffered saline
PFA	Paraformaldehyde
CTLS	Cleared Tissue Light Sheet
FG	Fast Green dye
BABB	Benzyl alcohol and benzyl benzoate
WC	Wound contraction
WD	Wound diameter
ECM	Extracellular matrix
SHG	Second harmonic generation
