# Supplementary material for: Different Retinoid Micellar Formulations on Wound Healing: Efficacy and Collagen Structure
Source: Pharmaceutics. 2026 Jun 9;18(6):708. doi: 10.3390/pharmaceutics18060708 (PMC13306382; doi:10.3390/pharmaceutics18060708)
Supplement: Supplementary file 1 [file pharmaceutics-18-00708-s001.zip › pharmaceutics-4292196-supplementary.pdf]

Supporting Information

2.1. Formulation of 0.3% Retinol Micelles

Micellar formulations containing 0.3% (w/v) retinol were prepared based on the method described in Phytoceutical Ltd IP, with minor modifications to optimise formulation performance. Key parameters including temperature, homogenisation speed, surfactant concentration, and solvent composition were systematically evaluated to achieve optimal solubilisation, encapsulation efficiency, and stability.

Optimised conditions were established using a Fisherbrand 850 Homogeniser operated at 5,000 rpm and a processing temperature of 50 °C. A solvent system comprising water and ethanol at a ratio of 10:1 (v/v) was employed, with 10% (w/v) tocopheryl polyethylene glycol 1000 succinate (TPGS) as the surfactant.

Briefly, 20 mL of prewarmed (50 °C) aqueous TPGS solution (10% w/v) was combined with 2 mL of ethanolic retinol solution (0.3% w/v). The mixture was maintained at 50 °C and subjected to high-shear homogenisation at 5,000 rpm for 30 minutes. During this process, partial evaporation of ethanol occurred, resulting in a final formulation volume of approximately 20 mL. The resulting system formed nano-sized micelles with improved solubilisation and physicochemical stability of retinol.

Following homogenisation, the formulation was allowed to cool to ambient temperature, transferred into amber scintillation vials, and stored under dark conditions to minimise photodegradation prior to further use.

2.2. Formulation of 0.3% Retinaldehyde Micelles

Micellar formulations containing 0.3% (w/v) retinaldehyde were prepared using the same optimised protocol described in Section 2.1. The only modification involved substituting retinol with retinaldehyde at an equivalent concentration (0.3% w/v) in ethanol. All other formulation parameters, including TPGS concentration, solvent ratio, temperature (50 °C), and homogenisation conditions (5,000 rpm, 30 minutes), were maintained constant to enable direct comparison between retinoid derivatives.

2.3. Formulation of 0.03% Retinoic Acid Micelles

Micellar formulations containing 0.03% (w/v) retinoic acid were prepared following the same procedure outlined in Section 2.1, with retinoic acid substituted in place of retinol.

The reduced concentration (0.03% w/v) was selected to reflect the higher biological potency of retinoic acid relative to its precursor retinoids.

All processing conditions, including TPGS concentration (10% w/v), solvent system (H_2O:EtOH, 10:1 v/v), homogenisation speed (5,000 rpm), and temperature (50 °C), were kept consistent to ensure comparability across formulations.
